# The diverse functions of Mu-class Glutathione S-transferase *HrGSTm1* during the development of *Hyalomma rufipes* with a focus on the detoxification metabolism of cyhalothrin

**DOI:** 10.1186/s13071-023-06084-6

**Published:** 2024-01-02

**Authors:** Meichen Zhao, Zhihua Gao, Xin Ji, Kuang Wang, Songbo Zhang, Yanqing Shi, Xuecheng Song, Zhijun Yu, Xiaolong Yang

**Affiliations:** Hebei Key Laboratory of Animal Physiology, Biochemistry and Molecular Biology, Hebei Collaborative Innovation Center for Eco-Environment, Ministry of Education Key Laboratory of Molecular and Cellular Biology, College of Life Sciences, Hebei Normal University, Shijiazhuang, 050024 China

**Keywords:** *Hyalomma rufipes*, Glutathione S-transferase, Prokaryotic expression, Enzyme activity, Functional analysis, Tick control

## Abstract

**Background:**

Glutathione S-transferases (GSTs) are a superfamily of multifunctional enzymes in living organisms with metabolic and detoxification functions, which can detoxify exogenous and endogenous compounds and thereby reduce the damage caused by toxic substances to the body. Ticks are obligate blood-sucking ectoparasites that can transmit various pathogens, and the characterization of tick-derived GSTs may help improve current understanding of the molecular mechanism of tick resistance to insecticides. In this study, a novel GST gene, named *HrGSTm1*, was identified from *Hyalomma rufipes*.

**Methods:**

Sequence analysis was performed by using bioinformatics techniques. A prokaryotic expression system was used to obtain the recombinant expression protein rHrGSTm1. Detection of spatiotemporal expression patterns of target genes and their response to the toxicity of cyhalothrin on female *H. rufipes* was performed by using a quantitative PCR platform. The optimal enzymological parameters of rHrGSTm1 using glutathione as substrate were calculated. The antioxidant capacity of the recombinant protein was evaluated by DPPH• (1,1-Diphenyl-2-picrylhydrazyl radical 2,2-Diphenyl-1-(2,4,6-trinitrophenyl) hydrazyl). Knockdown of the *HrGSTm1* genes through RNA interference was used to analyze their effects on the physiological parameters of ticks. The changes in *HrGSTm1* messenger RNA expression patterns under cypermethrin stress were analyzed.

**Results:**

The complementary DNA sequence of *HrGSTm1* contained a 672-bp open reading frame, which potentially encoded 223 amino acids. The predicted molecular weight was 25.62 kDa, and the isoelectric point 8.22. *HrGSTm1* is a Mu-class GST, belonging to the cytoplasmic GSTs with no signal peptide observed. The *V*_max_ and *K*_m_ of rHrGSTm1 were 3.367 ± 0.81 uM and 2.208 ± 0.76 uM, respectively, and its activities were dependent on different temperatures and pH conditions; the scavenging rate of rHrGSTm1 to DPPH• reached 76.4% at 1.25 mg/ml. Variable expressions of *HrGSTm1* were observed under various treatment periods and in different tissues, with the highest appearing in eggs (analysis of variance [ANOVA],* F*_(2, 9)_ = 279.9, *P* < 0.0001) and Malpighian tubules (ANOVA,* F*_(3, 12)_ = 290.5, *P* < 0.0001). After knockdown of *HrGSTm1*, compared with the control group, the mortality in the treatment group was increased by 16.7%, the average oviposition rate decreased by 33.9%, the average engorged body weight decreased by 287.38 mg and egg weight decreased by 127.46 mg, although only the engorged body weight was significantly different (t-test, *t*_(44)_ = 2.886, *P* = 0.006). After exposure to three sublethal concentrations (LC_05_, LC_10_, LC_50_) of cyhalothrin, the expression level of *HrGSTm1* in the midgut, ovary and salivary gland was upregulated, whereas in Malpighian tubules, it showed a trend of upregulation at first and then downregulation, implying different functions during the detoxification in different tissues.

**Conclusions:**

In this study, a novel GST of the Mu-class was successfully isolated from *H. rufipes* and systematically subjected to bioinformatic analysis and recombination identification. The variation trend of *HrGSTm1* expression level in different tissues suggests that the gene has different detoxification functions in different tissues. The potential function of this gene was analyzed to provide basic research for further investigation of its detoxification mechanism.

**Graphical Abstract:**

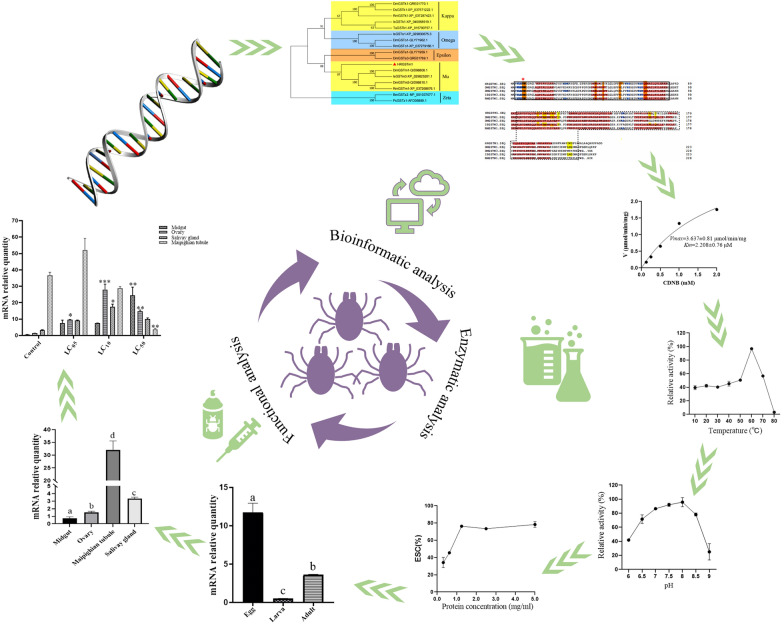

## Background

Glutathione S-transferases (GSTs) are an ancient superfamily of multifunctional enzymes in all living organisms. As major detoxifying enzymes, they can detoxify and metabolize exogenous toxic substances, and inactivate the endogenous α, β-unsaturated aldehydes, quinones, epoxides and the hydroperoxides produced during antioxidant or oxidative stress. In addition, they closely participate in the biosynthesis of leukotriene, prostaglandin, testosterone and progesterone and in the degradation of tyrosine [1]. GSTs are divided into cytosolic GSTs, mitochondrial GSTs and microsomal GSTs [2, 3]. The GST molecule is a homodimer with a molecular weight of about 50 kDa, and each monomer consists of an N-terminal domain folded by thioredoxin and a C-terminal domain with a helix connected by the connecting ring. The N-terminal domain of GSTs is relatively conservative, containing key specific residues for catalytic activity through the binding to the glutathione (GSH) through hydrogen bonds. Most of residues are tyrosine, cysteine or serine, which promote the deprotonation of GSH and produce active thiolation reactions. The variability of the C-terminal domain of GSTs enables these enzymes to use a variety of substrates to bind activated glutathione to most hydrophobic compounds containing electrophilic atoms [4].

As obligate blood-sucking ectoparasites, ticks are important vectors which can transmit a great diversity of pathogens, thus causing significant harm to humans and animals [5]. The tick *Hyalomma rufipes* mainly lives in desert or semi-desert areas, and often parasitizes domestic animals such as goats, cattle, horses and sheep. It can transmit diverse zoonotic pathogens, including *Babesia* spp., *Rickettsia* spp., Crimean-Congo hemorrhagic fever virus and human typhus fever virus [6].

Cyhalothrin is a synthetic type II pyrethroid insecticide extensively used for agricultural and domestic insect pest management all over the world [7]. Pyrethroids are usually recognized as sodium channel modulators which can modify the kinetics of voltage-gated sodium channels and inhibit various enzymes within insects and other pests [8]. However, GSTs cannot participate in the direct metabolism of pyrethroid insecticides, whereas they may mediate insect resistance to pyrethroid insecticides by detoxifying lipid peroxidation products induced by pyrethroids [9]. GSTs also protect organisms from toxicity by reducing the penetration of pyrethroids from the insect's body surface [10].

In the present study, a novel GST named *HrGSTm1* was cloned and identified in *H. rufipes*, followed by bioinformatic and enzymatic analysis. In addition, we further evaluated the antioxidant capacity, relative expression and detoxification function of this novel GST, in the hope of revealing the molecular mechanism of tick resistance to insecticides and providing the theoretical basis for subsequent tick control.

## Methods

### Collection and rearing of ticks

Unfed *H. rufipes* adults were collected from vegetation by flag-dragging in Wuhai county, Inner Mongolia Autonomous Region, northern China. The larvae and adults were raised on the ears of domestic rabbits (*Oryctolagus cuniculus*). After engorgement, the ticks were collected, placed in glass tubes containing one folded filter paper and stored in an incubator (26 ± 1 °C, 75 ± 5% relative humidity, 6:18-h light:dark cycle).

### Total RNA extraction and complementary DNA library construction

Groups of unfed adults (5 females and 5 males in each group) were collected, put into a precooled mortar and ground with liquid nitrogen into powder, then moved to a 1.5-ml EP tube for total messenger RNA (mRNA) extraction. The quality of the extracted RNA was assessed by spectrophotometry (NanoDrop® ND-1000; NanoDrop Technologies, Wilmington, DE, USA). The First-Strand cDNA Synthesis SuperMix Kit (TransGen Biotech, Beijing, China) was used to synthesize the first-strand complementary DNA (cDNA), following the manufacturer’s instructions. The cDNA was then stored at − 20 °C until use.

### Cloning and sequence analysis

Specific primers for *HrGSTm1* (forward primer 5′-ATGGCTCCGGTTCTCG-3′; reverse primer 5′-TCATGGCTTCTTCTGCAG-3′) were designed using Primer 5.0 software according to the GST sequence of *H. rufipes* from Connective Map (CMAP), and the PCR was carried out using specific primers. The PCR conditions were: 94 °C for 5 min; followed by 30 cycles each of 94 °C for 30 s, 58 °C for 30 s and 72 °C for 1 min; with a final elongation at 72 °C for 10 min. The PCR products were detected by agarose gel electrophoresis (1%) and sequenced. Sequence alignment was performed for *HrGSTm1* with other GST sequences from GenBank, and the physical and chemical parameters were analyzed. The maximum likelihood method was used, and the phylogenetic tree was constructed with Mega5 software.

### Prokaryotic expression system and protein purification of *HrGSTm1*

A prokaryotic expression system was used to obtain recombinant HrGSTml (rHrGSTm1). Briefly, the coding sequence (CDS) of *HrGSTm1* was cloned into the pET-32a(+) vector (TaKaRa, Shiga, Japan) with *Bam*HI and *Hin*dIII restriction sites, then transformed into competent *Escherichia coli* (DE3) cells (AxyGen, Shanghai, China) and cultured at 37 °C for 24 h. A single bacterial colony was selected for ordinary PCR and the positive clones were screened by sequencing.

The optimal expression conditions of rHrGSTm1 were selected to induce the expression. The bacterial cells with IPTG induction were centrifuged at 12,000 rpm for 10 min and rHrGSTm1 was extracted by ultrasonic fragmentation; bacteria without IPTG addition served as the negative control. The expression level of rHrGSTm1 in both the supernatant and precipitate was analyzed by sodium dodecyl sulfate–polyacrylamide gel electrophoresis (SDS-PAGE).

The rHrGSTm1 labeled with histidine (His) was purified by passage through an Ni column and eluted through an imidazole gradient of 20–500 mM; the purity was detected by SDS-PAGE. The rHrGSTm1 was identified by liquid chromatography-tandem mass spectrometry (LC–MS/MS) (Thermo Fisher Scientific, Waltham, MA, USA).

### Enzyme kinetics of rHrGSTm1

At the concentration of 5 mM GSH, the specific activity of rHrGSTm1 at different concentrations of 1-Chlom-2,4-Dinitrobenzene (CDNB, 0.125, 0.25, 0.5, 1 and 2 mM) were detected and calculated. For the negative control, the denatured recombinant protein boiled for 7 min was used as the substrate. Data were processed using GraphPad Prism 8.0 (GraphPad Software, San Diego, CA, USA) and the Michaelis constant (*K*_m_) and the maximum reaction rate (*V*_max_) of rHrGSTm1 were calculated by the Michaelis-Menten equation.

The formula used for the specific activity value was: specific activity (μmol/min/mg) = (ΔOD_340_ × V)/(ε × T × L × E), where ε is the molar extinction coefficient (9.6 mM/cm); E is the amount of enzyme added (1 μg); L, is the optical path (1 cm); ΔOD_340_ is the change of absorbance; V is the total reaction system (200 μl); and T is the reaction time (1 min).

### Effects of temperature and pH on the catalytic activity of rHrGSTm1

The catalytic activity of rHrGSTm1 was determined by water bath treatment at different temperatures (10 °C, 20 °C, 30 °C, 40 °C, 50 °C, 60 °C, 70 °C and 80 °C) for 15 min. Eight pH buffers (pH 6.0, 6.5, 7.0, 7.5, 8.0, 8.5 and 9.0) were used to detect the catalytic activity of rHrGSTm1, determined using phosphate buffers (100 mM) at pH 6.0, 6.5, 7.0, 7.5 and 8.0, and Tris hydrochloric acid buffers (100 mM) at pH 8.5 and 9.0. For the negative control, the denatured recombinant protein boiled for 7 min was used as the substrate. The experiment was repeated three times, and the highest enzyme activity was regarded as 100%.

### Antioxidant analysis

The 100× stock solution was prepared by dissolving 2.4 mg DPPH• in 1 ml of anhydrous ethanol and storing the solution in the dark. Before use, the stock solution was diluted with methanol to make a 6 × 10^–5^ DPPH• working solution; the rHrGSTm1 was diluted into five different concentrations (0.3125, 0.625, 1.25, 2.5 and 5.0 mg/ml). No substrate was added to the blank group (only ddH_2_O), whereas in the positive control group Trolox was added. The working solution and rHrGSTm1 were added to each well, and the absorbance were detected at 517 nm. Each sample was repeated three times.

$${\text{DPPH}} \cdot {\text{free radical scavenging rate}}:{\text{ ESC}}\% = \, \left[ {{1} - \left( {{\text{A}}_{{{\text{sample}}}} - {\text{A}}_{{{\text{blank}}}} } \right) \, /{\text{A}}_{{{\text{control}}}} } \right]*{1}00 \, \%$$

A_control_, the ratio of methanol to DPPH· (1:1); A_blank_, the ratio of rHrGSTm1and methanol was 1:1.

### Tissue distribution and stage-specific expression of *HrGSTm1*

The relative expression of *HrGSTm1* in different tissues and developmental stages was detected by Real-time Quantitative polymerase chain reaction (qPCR). As the first step, the primers for *HrGSTm1* (forward primer: 5′-CGGCTACTGGGACATTCG-3′; reverse primer: 5′-CACCGTCAATGTAGTAAGGCA-3′) and the primers for *β-Actin* as control (forward primer: 5’-CGTTCCTGGGTATGGAATCG-3′; reverse primer: 5′-TCCACGTCGCACTTCATGAT-3′) were designed. The ovary, Malpighian tubules, salivary gland and midguts of the engorged females were dissected in phosphate buffer, frozen with liquid nitrogen and stored at -80 °C, and three different developmental periods, including eggs, larvae and adults, were collected for subsequent assays.

Total RNA was extracted as described above, and the EasyScript® First-Strand cDNA Synthesis SuperMix Kit (TransGen, Beijing, China) was used to synthesize cDNA, following the manufacturer’s instructions. Then TransStart® Tip Green qPCR SuperMix Kit (TransGen) was used for the qPCR. Each sample was divided into an experimental group, actin control group and mixed sample group, and the qPCR was repeated three times. The mixed sample group was used to eliminate the background signals. The expression of *HrGSTm1* was measured by the 2^−ΔΔCt^ method, and the results of the qPCR assays were analyzed by GraphPad Prism 8.0 software (GraphPad Software). The results were analyzed by Student t-test and multivariate analysis of variance.

### Synthesis of double-stranded RNA in vitro and RNA interference

Specific primers (forward primer: 5′-TGGACGACAAGCGTTACTCAT-3′; reverse primer: 5′-CATAGGCAATGAAGTCAACATAAGT-3′) for *HrGSTm1* double-stranded RNA (dsRNA) and green fluorescent protein (GFP) (forward primer: 5′-TAATACGACTCACTATAGGG GACGTAAACGGCCACAAGT-3′; reverse primer: 5′-TAATACGACTCACTATAGGGGCTTCTCGTTGGGGTCTTT-3′) were designed using Primer version 5.0 software (GraphPad Software). After verified by PCR, the T7 RiboMAX^TM^Express RNAi system Kit (Promega, Madison, Wisconsin, USA) was used to synthesize dsRNA in vitro, following the manufacturer’s instructions. After synthesis, a Hamilton needle (Hamilton, Reno, NV, USA) was used for dsRNA injection of *H. rufipes*: 1 μl of dsRNA solution at the concentration of 4000 ng/μl was injected into the active female adults. Ticks were incubated in a laboratory incubator (27 ± 1 °C, relative humidity ≥ 80%) for 24 h and then placed on rabbit ears for blood-feeding. The physiological parameters, such as blood feeding time, engorgement body weight, fecundity and bite rate, were calculated and statistically analyzed [11].

### Expression response to chemical stress

The toxicity of cyhalothrin on female *H. rufipes* was determined by the impregnation method, and three sublethal concentrations (LC_05_, LC_10_ and LC_50_) of cyhalothrin were selected. The salivary glands, ovaries, midgut and Malpighian tubules of females that were treated with different sublethal concentrations were dissected as described above. Then the relative expression of *HrGSTm1* in different tissues was determined using qPCR. Ticks without cyhalothrin treatment were used as the negative control.

## Results

### Bioinformatics analysis

**Fig. 1:**
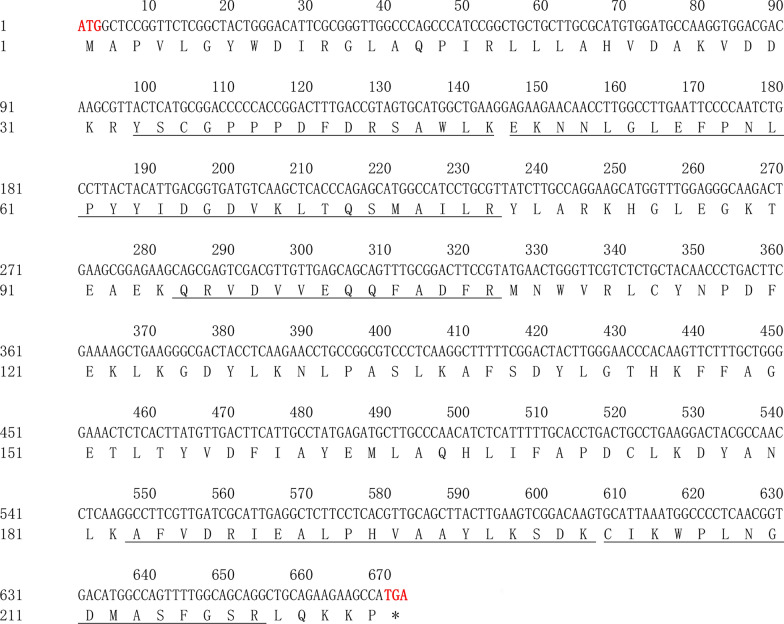
Amino acid sequence of the *HrGSTm1* gene. The ATG start codon and the TGA stop codon are marked in bold red font. The four unique peptides of the *HrGSTm1* gene are underlined

**Fig. 2:**
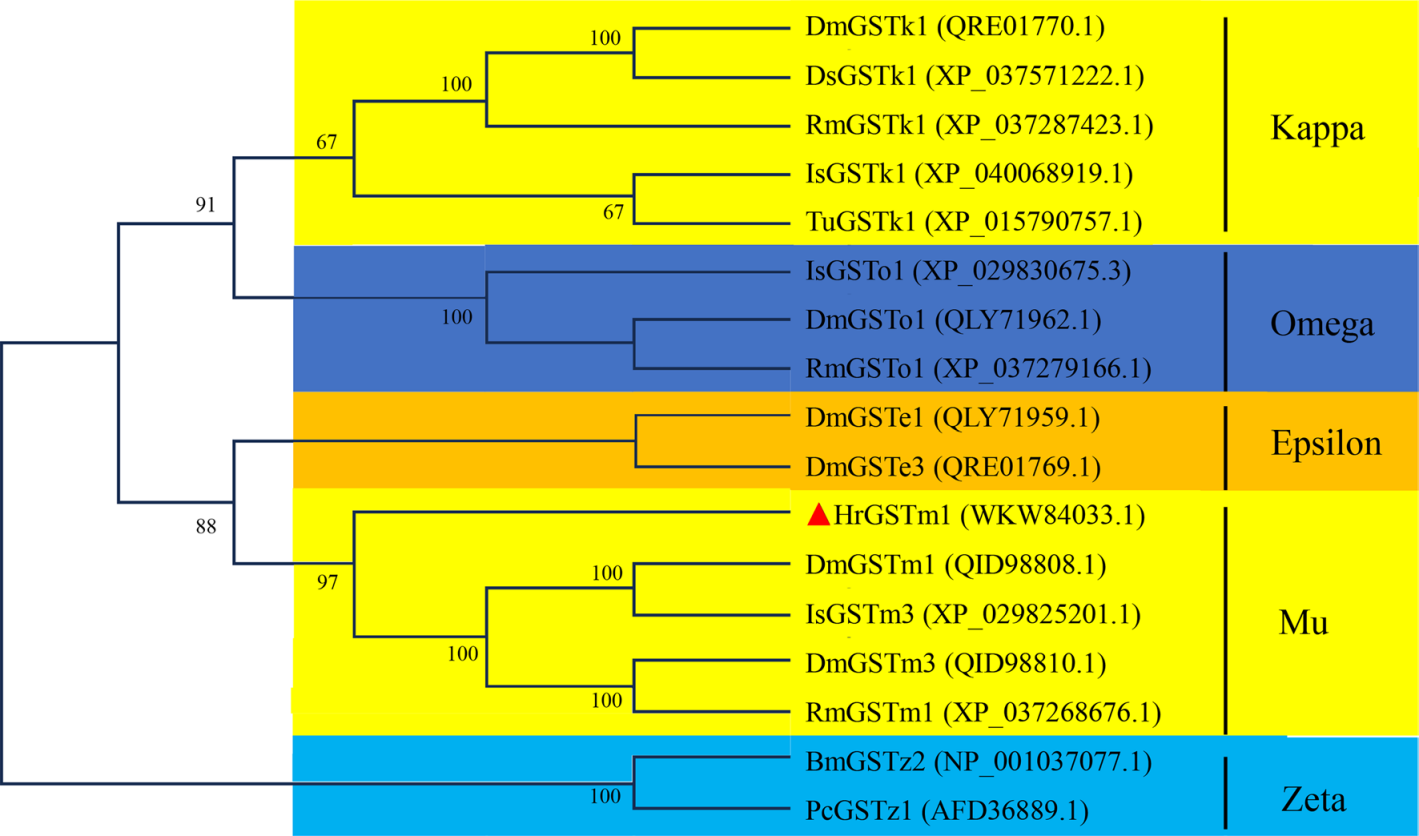
Phylogenetic tree of *HrGSTm1* with other species. Dm, * Dermacentor marginatus*; Hr,* Hyalomma rufipes*; Is,* Ixodes scapularis*; Pc, *Planococcus citri*; Rm, *Rhipicephalus microplus*; Ds, *Dermacentor silvarum*; Tu, *Tetranychus urticae*; Bm, *Bombyx mori*

The cDNA sequence of *HrGSTm1* (OQ790160) contains a 672-b open reading frame (ORF) that potentially encodes 233 amino acids (aa) (Fig. 1). The predicted molecular weight was 25.62 kDa, and the isoelectric point was 8.22. No signal peptide was predicted in *HrGSTm1*, which indicated that it is a cytoplasmic GST. The phylogenetic tree showed that the GSTs could be divided into five classes (Kappa, Omega, Epsilon, Mu, and Zeta), with the *HrGSTm1* of *H. rufipes* belonging to the Mu-class of GSTs (Fig. 2). Homologous sequence analysis showed that there was a sequence structure similar to a Mu loop at the position of 35-44 aa [12]. The GSTs of the same class have different branches. *DmGSTm1* (QID98808.1) of *Dermacentor marginatus* and *IsGSTm3-XP* (029825201.1) of *Ixodes scapularis* were clustered into a small branch; *DmGSTm3* (QID98810.1) of *D. marginatus* and *RmGSTm1-XP* (037268676.1) of *Rhipicephalus microplus* were clustered into a small branch; and *HrGSTm1* of *H. rufipes* and the upper two branches were clustered into one branch of the Mu-class.

**Fig. 3:**
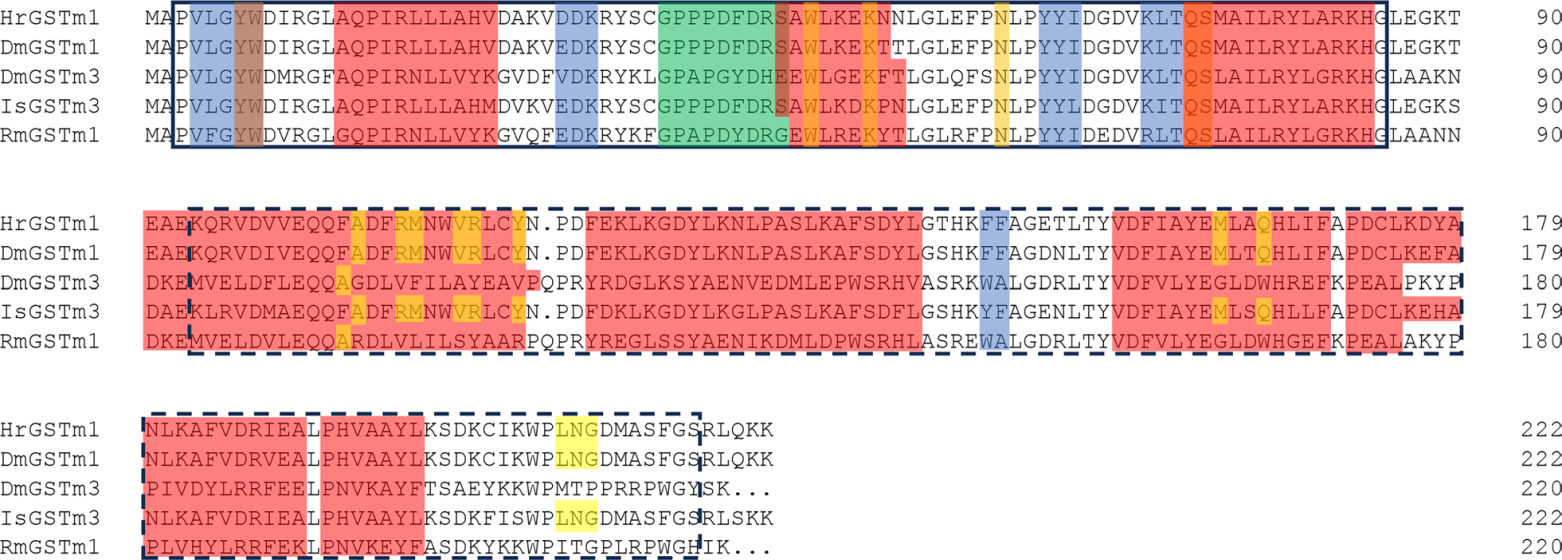
Alignment of the amino acid sequences between *HrGSTm1* with GSTs of other tick species in the Mu subfamily. Dots indicate catalytically active-site residues, the solid-line box indicates conserved N-terminal domains, the dashed-line box indicates the conserved C-terminal domain. The red area indicates* α*-helical element; the blue area,* β*-folded element; the yellow area, predicted G-sites; the light-yellow area, predicted H-sites; the green area, predicted Mu loop. Dm* Dermacentor marginatus*; GSTs, glutathione S-transferases; Hr,* Hyalomma rufipes*; Is,* Ixodes scapularis*; Rm,* Rhipicephalus microplus*

The protein encoded by *HrGSTm1* contained nine *α* helices and five *β* folds, and had conserved N-terminal and C-terminal domains. The N-terminal domain is in aa 3–83, which is characterized by the *βαβαββα* structure, a tyrosine catalytic residue site (Try7) and eight G-sites binding to GSH, namely Try7, Trp8, Trp45, Lys49, Asn58, Val59, Gln71 and Ser72. The C-terminal domain is in aa acid 93–124, which has six *α* structures, one *β* structure and 11 H-sites that bind to the substrate, namely Phe105, Ile108, Met109, Val112, Arg113, Tyr116, Ala165, Ile168, Ile211, Trp212 and Ser213 (Fig. 3).

### Prokaryotic expression and enzymatic characteristics of rHrGSTm1

**Fig. 4:**
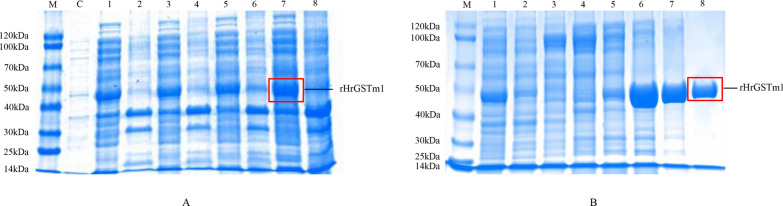
SDS-PAGE electrophoresis of purified proteins from rHrGSTm1. **a** SDS-PAGE of rHrGSTm1 in *Escherichia coli* BL21 under the optimized conditions. Lanes: M, Marker; C, bacteria without IPTG addition, swerving as the negative control; 1, 3, 5, 7, production of rHrGSTm1 in the supernatant with IPTG at 37 °C for 2, 4, 6 and 8 h, respectively; 2, 4, 6, 8, production of the rHrGSTm1 in the precipitation with IPTG at 37 °C for 2, 4, 6, 8, respectively. **b** SDS-PAGE of rHrGSTm1 eluted with different concentrations of imidazole. Lanes: M, Marker; 1, before affinity; 2, after affinity; 3–8, recombinant proteins eluted by 20, 50, 100, 200, 250 and 500 imidazole, respectively. SDS-PAGE, Sodium dodecyl sulfate-polyacrylamide gel electrophoresis; rHrGSTm1, recombinant HrGSTml protein

The molecular mass of rHrGSTm1 is 44 kDa, with the specific peptide (Table 1), indicating that the prokaryotic expression system of *E. coli* correctly expressed the GST proteins of *H. rufipes*. The expression of rHrGSTm1 was the highest in the supernatant of bacteria induced by 0.5 mM IPTG for 8 h at 37 °C, and a single band of rHrGSTm1 was obtained by elution with 500 mm imidazole (Fig. 4).

**Table 1:** Mass spectrometric identification of rHrGSTm1 protein

Protein	Unique peptides	Sequence	MH+ (Da)^a^	Proteins	*r* (cross correlation)
rHrGSTm1^a^	6	QRVDVVEQQFADFR	1736.87146	1	5.72
		CIKWPLNGDMASFGSR	1838.86763	1	3.79
		YSCGPPPDFDRSAWLK	1895.87449	1	3.92
		AFVDRIEALPHVAAYLKSDK	2243.21828	1	5.2
		EKNNLGLEFPNLPYYIDGDVKLTQSMAILR	3451.793	1	5.65

^a^Theoretical mass in Daltons

^b^Glutathione S-transferase of the Mu-class isolated from *Hyalomma rufipes*

**Fig. 5:**
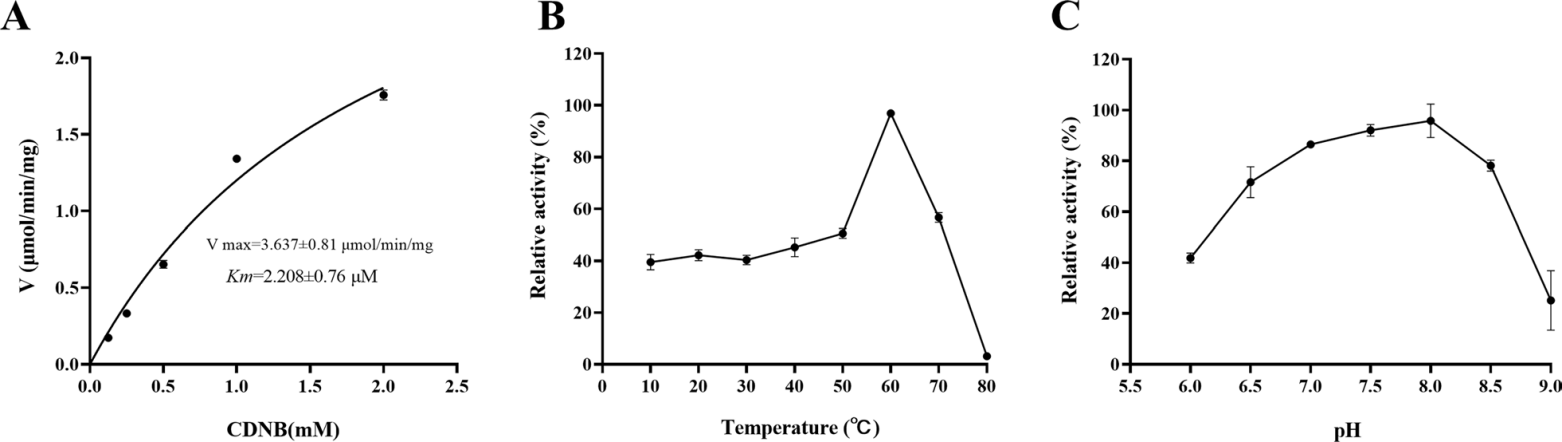
The enzymatic characteristics of the rHrGSTm1 protein. The denatured recombinant protein, boiled for 7 min, was used as the substrate. **a** Enzyme dynamics of the recombinant rHrGSTm1, **b** effect of temperature on enzyme activity, **c** effect of pH on enzyme activity. CDNB, 1-Chloro-2,4-dinitro-benzene; *K*_m_, Michaelis constant; *V*_max_, Maximum reaction rate; rHrGSTml, recombinant HrGSTml protein; V, reaction velocity

The *V*_max_ of rHrGSTm1 was 3.367 ± 0.81 μmol/min/mg, and the *K*_m_ was 2.208 ± 0.76 μM (Fig. 5a). The relative enzyme activity of rHrGSTm1 was stable at about 40% at 10–50 °C, increased with increasing temperature between 50 °C and 60 °C, reaching its peak activity at 60 °C, and decreased rapidly at higher temperatures, gradually becoming inactive at temperatures higher than 60 °C (Fig. 5b). Enzyme activity was barely affected at 10–50 °C, indicating that the protein structure was stable within this temperature range. The relative enzyme activity of rHrGSTm1 was the highest when pH 8.0, increased steadily when pH was 6.0–8.0, and decreased gradually when was pH 8.0–9.0 (Fig. 5C).

### Biological function analysis

**Fig. 6:**
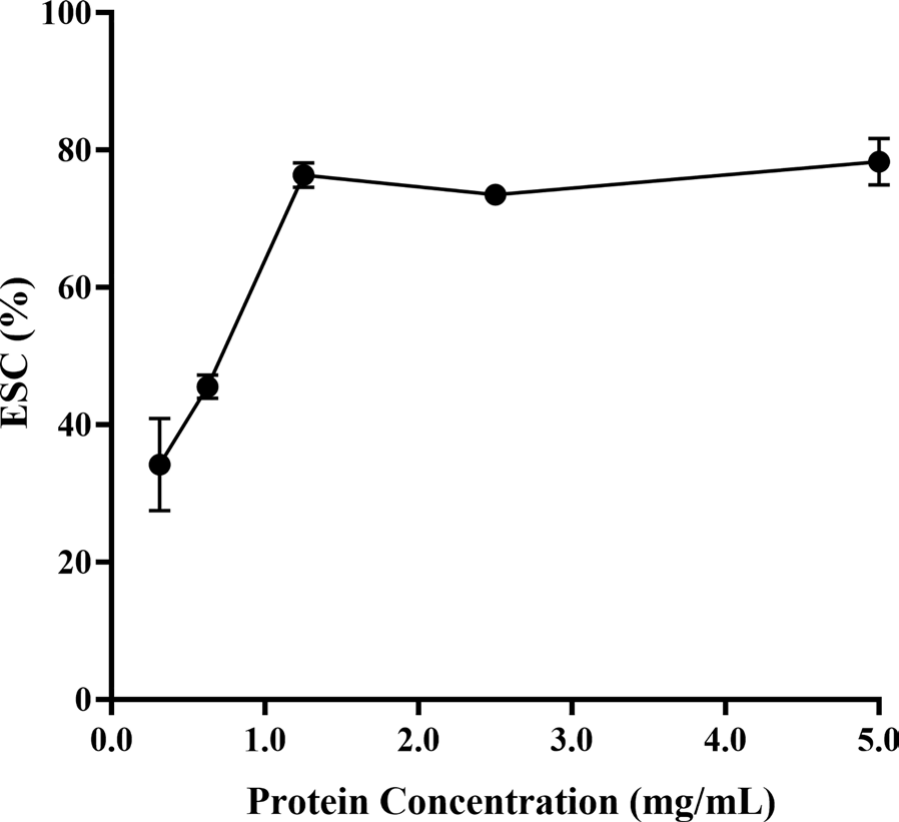
Detection of the free radical DPPH• scavenging ability of recombinant rHrGSTm1 protein. Water was used as the substrate in the blank control, and was Trolox was used as the substrate in the positive control. ESC, Ethylene scavenging capacity

The rHrGSTm1 had certain scavenging ability to DPPH•. The scavenging rate of rHrGSTm1 was 76.4% at 1.25 mg/ml, increased significantly with increasing protein concentration up to 1.25 mg/mL, but did not change significantly in the concentration range 1.25 to 5.0 mg/ml (Fig. 6).

**Fig. 7:**
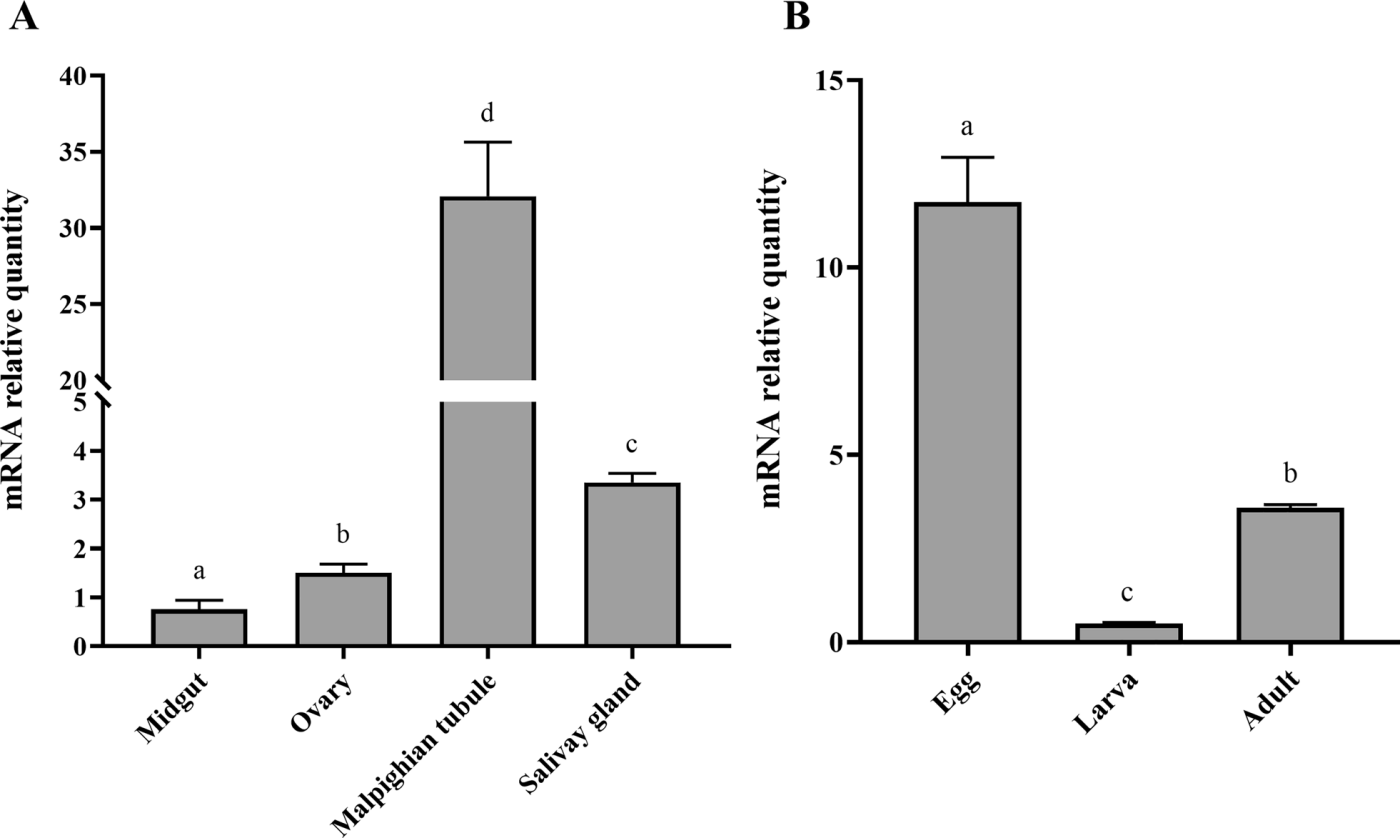
The spatio-temporal expression pattern of *HrGSTm1.* **a** Relative expression of *HrGSTm1* at different developmental stages. Samples were mixed to eliminate the background signals. **b** Relative expression of *HrGSTm1* in different tissues. Samples were mixed to eliminate the background signals. Different lowercase above bars indicate a significant difference at *P* < 0.05. mRNA, Messenger RNA

In terms of *HrGSTm1* expression in different developmental stages, *HrGSTm1* expression was highest in eggs, at about 23.29- and 3.27-fold higher than its expression in larvae and adults, respectively (analysis of variance [ANOVA], *F*_(2, 9)_ = 279.9, *P* < 0.0001) (Fig. 7a). *HrGSTm1* was highly expressed in Malpighian tubules and the salivary gland, followed by the ovary and midgut, and its expression was significantly higher in Malpighian tubules than in the other tissues (ANOVA,* F*_(3, 12)_ = 290.5, *P* < 0.0001) (Fig. 7b). After knockdown of HrGSTm1, compared with the control group, the mortality in the treatment group was increased by 16.7%, the average oviposition rate decreased by 33.9%, the average engorged body weight decreased by 287.38 mg and the weight of eggs decreased by 127.46 mg; only engorged body weight was significantly different (t-test,* t*_(44)_ = 2.886, *P* = 0.006). (Table 2).

**Table 2:** Effects of RNA interference of *HrGSTm1* on biological traits of *Hyalomma rufipes*

Effects	Control group	Experimental group
Total number of ticks	18	18
Average mortality (%)	27.78%a	44.44%a
Mean spawning rate (%)	53.85%a	20.00%a
Engorged weight (mg)	853.04 ± 85.48a	565.66 ± 41.01b
Oviposition (mg)	447.41 ± 60.84a	319.95 ± 52.95a
Period of blood-feeding (days)	10.7a	11.5a

Values (mean ± standard deviation, where indicated) followed by different lowercase letters represent significant differences at *P* < 0.05 between groups

**Fig. 8:**
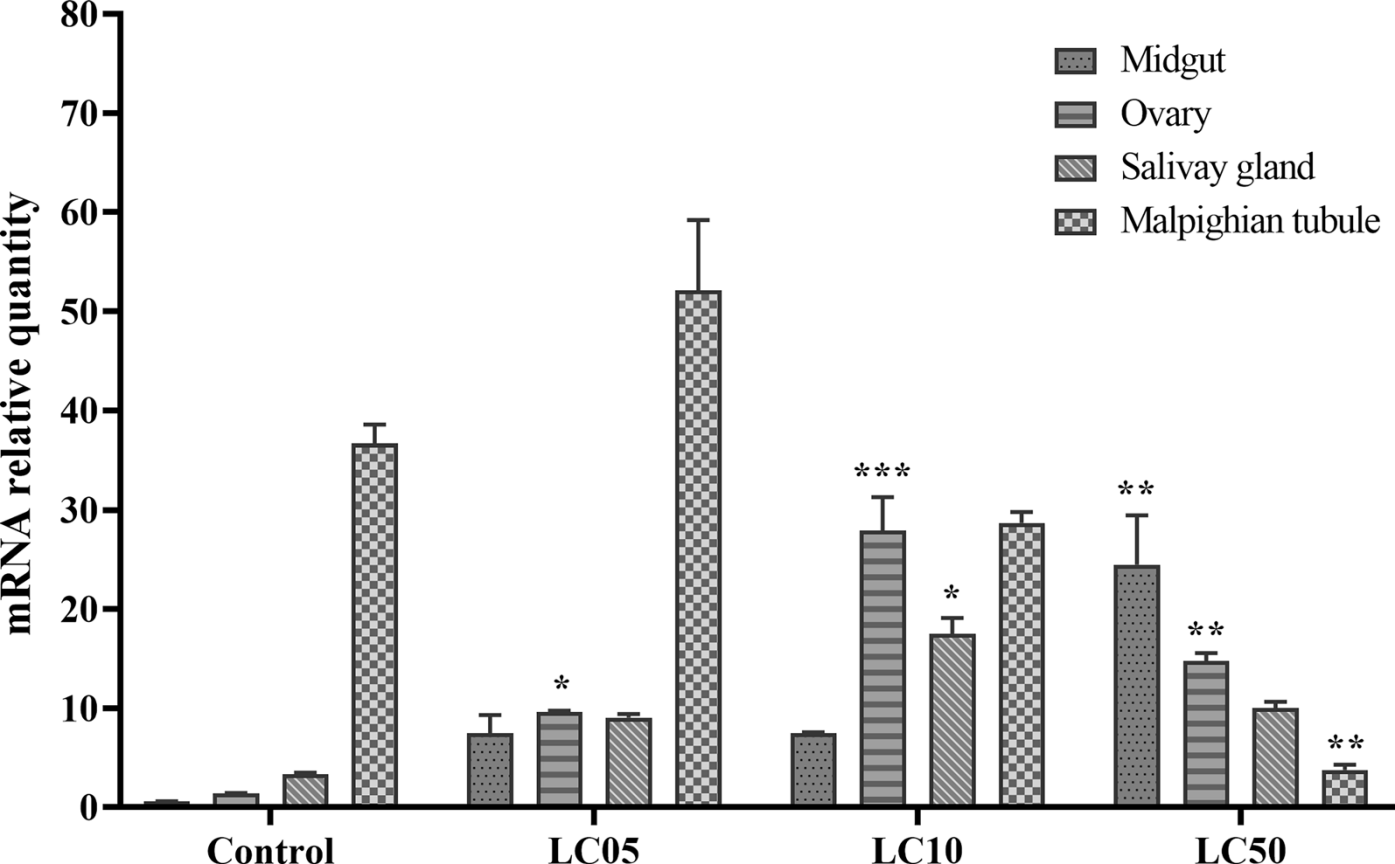
Effect of different concentrations of cypermethrin on *HrGSTm1* expression in different tissues of *H. rufipes*. Asterisks indicate significant differences at *P < 0.05, **P < 0.01 and ***P < 0.001). Ticks without cyhalothrin treatment were used as the negative control. LC_05_, LC_10_, LC_50_, Sublethal concentrations

 The relative expression of *HrGSTm1* in the midgut increased significantly with increasing sublethal concentration of cyhalothrin (ANOVA,* F*_(3, 12)_ = 58.87, *P* < 0.0001), and its relative expression was highest in the LC_50_ treatment. The relative expression of *HrGSTm1* in the ovary was 6.84-, 19.84- and 10.49-fold higher than that in the control group, and its relative expression was highest in the LC_10_ treatment (ANOVA,* F*_(3, 12)_ = 166.9, *P* < 0.0001). The relative expression of *HrGSTm1* in salivary glands was 2.71-, 5.22- and 2.99-fold higher than that in the control group, and its relative expression was highest in LC_10_ treatment (ANOVA,* F*_(3, 12)_ = 178.8, *P* < 0.0001), which was 1.75-fold higher than that in the LC_05_ and LC_50_ treatments. The relative expression in Malpighian tubules was the highest in the LC_05_ treatment (ANOVA,* F*_(3, 12)_ = 117.1, *P* < 0.0001), and that in LC_10_ and LC_50_ decreased by 1.87- and 13.18-fold, respectively (Fig. 8).

## Discussion

*HrGSTm1* contains a 672-bp ORF, and no signal peptide was predicted in *HrGSTm1*, indicating that it belongs to the family of cytoplasmic GSTs. Similarly, no signal peptide was found in *DvGST1* and *DvGST2* of *Dermacentor variabilis*, which also belong to the family of cytoplasmic GSTs [13]. The predicted N-terminal domain structure of *HrGSTm1* was *βαβαββα*, a thioredoxin structure with G-site, which is consistent with *McGST1* of *Mytilus coruscus* and *CsGSTM1* of *Clonorchis sinensis* [14–16]. A tyrosine catalytic residue (Tyr7) typical of the Mu-subfamily is also present in the N-terminal domain of *HrGSTm1*, which suggests that this residue can catalyze the binding of genes to GSH. In the general case, the GSH binding site consists of a stable tyrosine catalytic residue (Try), which plays a key role in the construction of three-dimensional structure [15]. The tyrosine residues of Mu-class GSTs of different species are differ with respect to location, with the Mu-class GST of *Penaeus monodon* (*PmMuGST*) located at position 7 (Tyr7) [17], and those in marine mollusks *Mytilus coruscus* (*McGST1*) and *Ruditapes philippinarum* (*RpGSTμ*) located at position 6 (Tyr6) [15, 18].

The rHrGSTm1 showed a certain degree of antioxidant activity and a certain scavenging ability to DPPH•. It has been proven that lipid peroxidation level increases in the egg of *Haemaphysalis longicornis*, indicating that these ticks may adjust redox balance to maintain survival when levels of reactive oxygen species increase or when they encounter oxidative stress [19]. In addition, some insects, such as *Apis cerana*, *Spodoptera exigua* and *Bombus ignites*, can also initiate the corresponding oxidative stress mechanism when, for example, they encounter heavy metals and toxic compounds [20–22].

The *V*_max_ of rHrGSTm1 was lower than that of *H. longicornis* rHlGST1 and rHlGST2 (*V*_max_ = 11.70 ± 1.92 and 14.72 ± 0.56 μmol/min/mg, respectively) [23] and higher than that of *Bombyx mori* rBmGSTD (*V*_max_ = 0.531 μmol/min/mg) and *Locusta migratoria* LmGSTs3 (*V*_max_ = 1.39 ± 0.20 μmol/min/mg) [24]. The *K*_m_ of rHrGSTm1 was lower than that of *Planococcus citri* rPcGSTd2 (*K*_m_ = 3.23 ± 1.02 μM) and *P. citri* rPcGSTd3 (*K*_m_ = 3.55 ± 1.12 μM), indicating that the affinity of rHrGSTm1 to the substrate was stronger than that of rPcGSTd2 and rPcGSTd3 [25]. The thermal stability profile showed that the optimum temperature of rHrGSTm1 was about 60 °C and that the enzyme activity was stable at temperatures lower than 50 °C, which is similar to the thermal stability of *P. citri* GSTs [25]. The stability of the pH showed that rHrGSTm1 had high catalytic activity when the pH was in the range 6.5–8.5, which is roughly the same as the optimum pH of *Boophilus annulatus* Mu GSTs and *Tigriopus japonicus* GSTs [26, 27].

The relative expression of *H. rufipes HrGSTm1* was found to clearly change under various treatment conditions and in different tissues. *HrGSTm1* relative expression was significantly higher in eggs than in larvae and adults. Similarly, the expression of *H. longicornis* GSTs increased significantly during embryonic development, with the highest expression on the first day after spawning, the early stage of embryogenesis and the 10th day when the germ band could be observed [19]. Some insect GSTs have also been found to be significantly higher in the egg and larval stages than in the adult stage, as observed in *Zeugodacus cucurbitae* and *Bactrocera dorsalis* [28, 29]. In terms of expression in different organs, the relative expression of *HrGSTm1* was highest in the Malpighian tubules, likely related to the function of the Malpighian tubules as the main excretory organ of waste. Similarly, the high expression of *GSTd3* in Malpighian tubule of *Cydia pomonella* has also been demonstrated to be involved in the metabolism of toxic chemicals [30].

After RNA interference (RNAi) of *HrGSTm1*, the mortality and feeding duration increased, while oviposition rate, weight of eggs laid and engorged body weight decreased, although only the engorged body weight was significantly different (t-test,* t*_(44)_ = 2.886, *P* = 0.006). A similar significant increase in mortality was also observed after RNAi-mediated knockdown of GST in *Z. cucurbitae* [28]. According to the experimental results, GST has an important effect on the blood-sucking behavior of ticks, which is consistent with previous results [23]. However, there are several limitations associated with the RNAi [31]. Due to the half-life of the dsRNA, the effectiveness of RNAi gradually weakens with an increasing length of time after treatment, so there may be a discrepancy in the life events (such as laying and hatching of eggs) after engorgement of ticks. Additionally, off-target effects and a low efficiency of RNAi have also been observed in ticks [32, 33]. Although micro-injection of dsRNA for RNA silencing is commonly used in ticks, the irreversible mechanical damage may also affect the development of ticks. Hence, it is necessary to identify an appropriate approach by which to efficiently knockdown the target gene without causing mechanical damage to ticks.

The expression of *HrGSTm1* in the midgut, ovary, salivary gland and Malpighian tubule changed significantly following exposure to different sublethal concentrations of cyhalothrin. However, due to post-transcriptional and post-translational regulation, changes in the transcription level may not completely correspond to the changes in protein level. Unfortunately, in the current study, the relative expression of the HrGST protein was not confirmed at the protein level due to the lack of specific anti-GST antibody in *H. rufipes*. It has been demonstrated that the increased activity of GSTs could result from gene amplification at the DNA level or overexpression at the mRNA level [10].

Previous studies have found that drug stress can induce or inhibit the expression of GSTs [24]. For example, the expression of *Nilaparvata lugens NLGSTE* decreased by 36% after exposure to 0.1 mM aldicarb [34]; the expression of *SLGSTe2* and *SLGSTe3* of *Spodoptera litura* was slightly upregulated after exposure to carbaryl, DDT and deltamethrin, but neither of these could be induced after exposure to malathion [35]; and the expression of *Dugesia japonica* GSTs was significantly inhibited after exposure to high concentration of glyphosate for 5 days [36]. In the present study, the expression of *HrGSTm1* in the Malpighian tubules was higher than that in the midgut. Previous studies have shown that the efficient detoxification rate occurs in tissues with a high metabolic rate, such as the Malpighian tubules and fat body in insects, while the Malpighian tubule is the main organ for the excretion of metabolic waste in the body [37]; these findings are is consistent with the results of the present study. Hence, we speculate that *HrGSTm1* may be involved in the detoxification metabolism of cyhalothrin in H. rufipes.

According to the results of this study, the knockdown of *HrGSTm1* had a significant effect on blood-sucking behavior, which may be the reason for the decline of other physiological parameters. Under the various stress conditions introduced by exposure to cyhalothrin, *HrGSTm1* in different tissues showed different regulatory trends, suggesting that ticks may have multiple strategies to cope with toxic substances.

This study provides basic knowledge that can be applied for developing candidate antigens of anti-tick vaccines and for improving the metabolic process of tick detoxification. The findings of the present study support the notion that GSTs is involved in the process of ticks’ resistance against toxic compounds and can facilitate further analysis on the detoxification and metabolism of GSTs in ticks to acaricides, thus providing a theoretical foundation for further study of tick resistance and providing strategies for chemical control of ticks.

## Conclusions

A novel GST of the Mu-class was isolated from *H. rufipes*, and the expression level of *HrGSTm1* was found to vary among different tissues, implying that the gene has distinct detoxification functions specific to each tissue. The rHrGSTm1 showed a certain degree of antioxidant activity and a certain scavenging ability to DPPH•. The potential function of this gene was analyzed to lay the groundwork for future research aimed at investigating its detoxification mechanism.

## Data Availability

The data that support the findings of this study are available from the database of NCBI and the corresponding author upon reasonable request. The sequence of *HrGSTm1* has been uploaded to GenBank and its accession number is OQ790160.

## References

[1] Hayes JD, Flanagan JU, Jowsey IR (2005). Glutathione transferases. Annu Rev Pharmacol Toxicol.

[2] Liu J, Yang X, Zhang Y (2014). Characterization of a lambda-cyhalothrin metabolizing glutathione S-transferase CpGSTd1 from *Cydia pomonella* (L.). Appl Microbiol Biotechnol.

[3] Gao SS, Li DY, Huo ZK, Zhang YL, Cao YZ, Tan YY (2022). A sigma class glutathione S-transferase gene regulated by the CncC pathway is required for phytochemical tolerance in the red flour beetle, *Tribolium castaneum*. J Asia-Pacific Entomol.

[4] Valenzuela-Chavira I, Contreras-Vergara CA, Arvizu-Flores AA (2017). Insights into ligand binding to a glutathione S-transferase from mango: structure, thermodynamics and kinetics. Biochimie.

[5] Estrada-Peña A (2015). Ticks as vectors: taxonomy, biology and ecology. Rev Sci Tech.

[6] Deng GF, Jiang ZJ. Economic entomology of China, 39. Acariidae: Ixodidae. Insect Knowledge. 1992;54.

[7] Fetoui H, Makni M, Garoui EM, Zeghal N (2010). Toxic effects of lambda-cyhalothrin, a synthetic pyrethroid pesticide, on the rat kidney: involvement of oxidative stress and protective role of ascorbic acid. Exp Toxicol Pathol.

[8] Van Dyk JS, Pletschke B (2011). Review on the use of enzymes for the detection of organochlorine, organophosphate and carbamate pesticides in the environment. Chemosphere.

[9] El-Demerdash FM (2011). Lipid peroxidation, oxidative stress and acetylcholinesterase in rat brain exposed to organophosphate and pyrethroid insecticides. Food Chem Toxicol.

[10] Enayati AA, Ranson H, Hemingway J (2005). Insect glutathione transferases and insecticide resistance. Insect Mol Biol.

[11] Gao Z, Zheng P, Wang K, Ji X, Shi Y, Song X (2022). The molecular and functional characterization of ferritins in the hard tick *Hyalomma rufipes*. Parasit Vectors.

[12] Hearne JL, Colman RF (2006). Contribution of the mu loop to the structure and function of rat glutathione transferase M1–1. Protein Sci.

[13] Dreher-Lesnick SM, Mulenga A, Simser JA, Azad AF (2006). Differential expression of two glutathione S-transferases identified from the American dog tick, *Dermacentor variabilis*. Insect Mol Biol.

[14] Sun YJ, Kuan IC, Tam MF, Hsiao CD (1998). The three-dimensional structure of an avian class-mu glutathione S-transferase, cGSTM1–1 at 1.94 A resolution. Mol Biol.

[15] Liu H, He J, Zhao R, Chi C, Bao Y (2015). A novel biomarker for marine environmental pollution of pi-class glutathione S-transferase from *Mytilus coruscus*. Ecotoxicol Environ Saf.

[16] Hong SJ, Lee JY, Lee DH, Sohn WM, Cho SY (2001). Molecular cloning and characterization of a mu-class glutathione S-transferase from *Clonorchis sinensis*. Mol Biochem Parasitol.

[17] Wang Y, Liu L, Huang J (2016). Response of a Mu-class glutathione S-transferase from black tiger shrimp *Penaeus monodon* to aflatoxin B1 exposure. Springerplus.

[18] Bathige SD, Umasuthan N, Saranya Revathy K, Lee Y, Kim S, Cho MY (2014). A mu class glutathione S-transferase from Manila clam *Ruditapes philippinarum* (RpGSTμ): cloning, mRNA expression, and conjugation assays. Comp Biochem Physiol C Toxicol Pharmacol.

[19] Hernandez EP, Shimazaki K, Niihara H, Umemiya-Shirafuji R, Fujisaki K, Tanaka T (2020). Expression analysis of glutathione S-transferases and ferritins during the embryogenesis of the tick *Haemaphysalis longicornis*. Heliyon.

[20] Yan H, Meng F, Jia H, Guo X, Xu B (2012). The identification and oxidative stress response of a zeta class glutathione S-transferase (GSTZ1) gene from *Apis cerana* cerana. Insect Physiol.

[21] Xu P, Han N, Kang T, Zhan S, Lee KS, Jin BR (2016). SeGSTo, a novel glutathione S-transferase from the beet armyworm (*Spodoptera exigua*), involved in detoxification and oxidative stress. Cell Stress Chaperones.

[22] Kim Y, Cha SJ, Choi HJ, Kim K. Omega class glutathione S-transferase: antioxidant enzyme in pathogenesis of neurodegenerative diseases. Oxid Med Cell Longev. 2017;2017:5049532. 10.1155/2017/5049532. 10.1155/2017/5049532 PMC5757135 29435097

[23] Hernandez EP, Kusakisako K, Talactac MR (2018). Characterization and expression analysis of a newly identified glutathione S-transferase of the hard tick *Haemaphysalis longicornis* during blood-feeding. Parasit Vectors.

[24] Qin G, Jia M, Liu T (2012). Heterologous expression and characterization of a sigma glutathione S-transferase involved in carbaryl detoxification from oriental migratory locust, *Locusta migratoria* (Meyen). Insect Physiol.

[25] Liao CY, Xia WK, Feng YC, Li G, Liu H, Dou W (2016). Characterization and functional analysis of a novel glutathione S-transferase gene potentially associated with the abamectin resistance in *Panonychus citri* (McGregor). Pestic Biochem Physiol.

[26] Shahein YE, El Sayed E-H, Abouelella AM, Hamed RR, Allam SA, Farid NM (2008). Molecular cloning, expression and characterization of a functional GSTmu class from the cattle tick *Boophilus annulatus*. Vet Parasitol.

[27] Lee YM, Lee KW, Park H (2007). Sequence, biochemical characteristics and expression of a novel Sigma-class of glutathione S-transferase from the intertidal copepod, *Tigriopus japonicus* with a possible role in antioxidant defense. Chemosphere.

[28] Ma M, Zhai XD, Xu HQ, Guo PY, Wang JJ, Wei D (2023). Genome-wide screening and expression of glutathione S-transferase genes reveal that GSTe4 contributes to sensitivity against β-cypermethrin in *Zeugodacus cucurbitae*. Int J Biol Macromol.

[29] Hu F, Dou W, Wang JJ, Jia FX, Wang JJ (2014). Multiple glutathione S-transferase genes: identification and expression in oriental fruit fly, *Bactrocera dorsalis*. Pest Manag Sci.

[30] Wei ZH, Liu M, Hu C, Yang XQ (2020). Overexpression of glutathione S-transferase genes in field λ-cyhalothrin-resistant population of *Cydia pomonella*: reference gene selection and expression analysis. J Agric Food Chem.

[31] Marr EJ, Sargison ND, Nisbet AJ, Burgess STG (2014). RNA interference for the identification of ectoparasite vaccine candidates. Parasite Immunol.

[32] Lew-Tabor AE, Kurscheid S, Barrero R, Gondro C, Moolhuijzen PM, Valle MR (2011). Gene expression evidence for off-target effects caused by RNA interference-mediated gene silencing of Ubiquitin-63E in the cattle tick *Rhipicephalus microplus*. Int J Parasitol.

[33] Zhang Y, Cui J, Zhou Y, Cao J, Gong H, Zhang H (2018). Liposome mediated double-stranded RNA delivery to silence ribosomal protein P0 in the tick *Rhipicephalus haemaphysaloides*. Ticks Tick Borne Dis.

[34] Saruta F, Yamada N, Yamamoto K (2019). An omega-class glutathione S-transferase in the brown planthopper *Nilaparvata lugens* exhibits glutathione transferase and dehydroascorbate reductase activities. Arch Insect Biochem Physiol.

[35] Deng H, Huang Y, Feng Q, Zheng S (2009). Two epsilon glutathione S-transferase cDNAs from the common cutworm, *Spodoptera litura*: characterization and developmental and induced expression by insecticides. Insect Physiol.

[36] Zhang HC, Yang YJ, Ma KX, Shi CY, Chen GW, Liu DZ (2020). A novel sigma class glutathione S-transferase gene in freshwater planarian *Dugesia japonica*: cloning, characterization and protective effects in herbicide glyphosate stress. Ecotoxicology.

[37] Qin G, Jia M, Liu T (2011). Identification and characterisation of ten glutathione S-transferase genes from oriental migratory locust, Locusta *migratoria manilensis* (Meyen). Pest Manag Sci.

